# Per oral substitution with 300000 IU vitamin D (Cholecalciferol) reduces bone turnover markers in HIV-infected patients

**DOI:** 10.1186/1471-2334-13-577

**Published:** 2013-12-06

**Authors:** Rein Jan Piso, Madeleine Rothen, Jean Pierre Rothen, Matthias Stahl, Christoph Fux

**Affiliations:** 1 Department of Medicine, Kantonsspital, Olten, Switzerland; 2 Department of Medicine, Regionalspital, Biel, Switzerland; 3 Medical Laboratories Olten, Olten, Switzerland; 4 Departement of Medicine, University Hospital, Aarau, Switzerland

## Abstract

**Background:**

Osteoporosis and bone fractures seem to be higher in HIV-infected Patients compared to the general populations. Moreover, bone turnover markers are increased in patients on antiretroviral therapy and vitamin D deficiency is prevalent in HIV-infected patients. However, the influence of per oral cholecalciferol on bone metabolism in HIV infected patients is not well understood.

**Methods:**

We measured the bone turnover markers in 96 HIV-infected patients: Bone specific alkaline phosphatase (BSAP), Pyridinoline (PYR), Desoxypyridinoline (DPD) and 25-OH vitamin D. If 25-OH vitamin D was below 75 nnol/L (87/96 patients), 300000 IU cholecalciferol was given per os. 25OH-vitamin D and bone turn over markers were determinded 3 month later. 25 OH-vitamin D was corrected for circannual rythm $\left[y^{'}=y+17.875*\sin\;\left(2\frac{\pi }{365}*\mathrm{day}+2.06\right)\right]$, whereas bone turnover markers were not corrected. The paired students t-Test was used to compare the two periods. No calcium supplementation or biphosphonate therapy was given.

**Results:**

Corrected 25OH-vitamin D levels increased significantly after supplementation (42.7 ± 26.61 vs. 52.85 ± 21.8 nmol/L, p < 0.001). After supplementation, bone turnover markers were significantly lower. The values decreased for BSAP from 21.31 ± 14.32 to 17.53 ± 8.17 μg/L (p < 0.001), PYR from 74.57 ± 36.83 to 54.82 ± 21.43 nmol/mmol creatinine (p < 0.001) and DPD from 15.17 ± 8.34 to 12.61 ± 5.02 nmol/mmol creatinine (p = 0.01).

**Conclusions:**

After per oral substitution with cholecalciferol, bone formation as well as bone resorption markers decreased significant. We postulate a protective effect on bone structure with cholecalciferol supplementation.

## Background

With the decline of opportunistic infections as consequence of antiretroviral therapy, life expectancy of HIV infected patients has prolonged remarkably [1]. In this newer era, in which HIV-infection is considered as a chronic, treatable disease, the focus on long time side effects of antiretroviral treatment as well as issues related to aging, partly aggravated by the HIV-infection itself, will become more and more important.

Recognized as a growing epidemic in the older, non HIV infected population, osteoporosis has become of particular interest to physicians treating HIV patients as well [2].

In HIV patients, an increased incidences of bone fractures has been observed [3], and it has been suggested that vitamin D deficiency [4,5] and low bone mineral density [6,7] are important pathogenic factors. Efavirenz has been shown to decrease vitamin D levels [5,8], and antiretroviral treatment was associated with increased bone turnover [9] as well as decreases in bone mineral density [10,11].

Levels of vitamin D are influenced by sunlight exposure and follow a seasonal rhythm [12]. Supplementation of low vitamin D levels has been widely adopted in HIV-care, but the benefits with respect to bone turn over, bone mineral density or fracture rate has not been clearly demonstrated. Most studies in non-HIV patients with osteoporosis have combined cholecalciferol and calcium supplementation [13,14]. A decrease in bone resorption as well as preservation of bone mineral density has been observed with a combination of cholecalciferol and calcium, but not with calcium supplementation alone [15].

Low levels of vitamin D in HIV-patients compared to the general population are well described. However it is still not understood if this is due to the HIV infection itself or to traditional risk factors. Therefore, we compared vitamin D levels not with the general population, but with HBV and HCV monoinfected patients, postulating similar living conditions as well as chronic inflammatory status. Moreover, osteoporosis and low vitamin D level has also described in hepatitis B and C patients [16-19]. 25OH-vitamin D levels may also influence treatment outcome in Hepatitis C patients [20].

In patients with low 25OH-vitamin D levels, we supplemented cholecalciferol and controlled bone turnover markers before and after supplementation. We choose a high dose pulse therapy primarily to limit pill burden, as it can be given at visit in the outpatient clinic.

## Methods

Patients were recruited in the outpatient infectious disease clinic of Kantonsspital Olten. Baseline characters are presented in Tables 1 and 2. Patients were excluded if a start or switch in ART had been performed in the last six months, as this may interfere with 25OH-Vitamin D levels and bone turnover. In patients with urgent need of antiretroviral therapy, no intervention data were collected for the same reason. Patients with Hepatitis monoinfection were excluded if treated with interferon. Dexa scans and nutrional data collection were not performed. At the follow up visit, questionnaire concerning overall physical health including specific questioning pain (bone fractures, nephrolitiasis) or nausea was filled out. Blood testing including liver, renal, metabolic and immunological parameters was also performed.

**Table 1:** Baseline characteristics HIV patients

	**ART (%)**	**No ART(%)**	**p-value**	**TDF (%)**	**No TDF(%)**	**p-value**	**PI**	**NNRTI**	
**Men**	40 (54)	15 (65)	0.38	26 (48)	14 (73)	p = 0.054	21 (65)	17 (42)	0.03
**Women**	33 (36)	8 (35)		28 (52)	5 (27)		10 (35)	23 (58)	
**Total**	73 (76)	23 (24)		54 (74)	19 (26)		32 (44)	40 (56)	
**Age ± SD**	43 ± 9	43 ± 9	0.74	42 ± 10	43 ± 11	p = 0.84	44 ± 9	41 ± 8	p = 0.08
**CD4 < 200**	10 (50)	10 (50)	0.003	6 (60)	4 (40)	p = 0.4	6 (60)	4 (40)	p = 0.3
**CD4200-350**	23 (92)	2 (8)		16 (76)	5 (24)		7 (30)	15 (70)	
**CD4 > 350**	40 (78)	11 (22)		32 (80)	8 (20)		18 (45)	21 (55)	

**Table 2:** Baseline characteristics

	**HIV (%)**	**Hepatitis monoinfection (%)**	**p-value**
**Men**	55 (57)	41(57)	0.96
**Women**	41 (43)	31 (43)	
**Total**	96	72	
**Age**	43 ± 9	43 ± 12	0.75
**Opiat consumption/ substitution**	31 (32)	23 (32)	0.82
**Diabetes mellitus**	5 (5)	2 (3)	0.65
**HCV infection**	36 (37)	50 (69)	<0.001

25OH-vitamin D, bone specific alkaline phosphatase (BASP) were measured in serum, pyridinoline (PYR) and desoxypyrindinoline (DPD) crosslinks were measured in first morning urine. Patients with 25OH-vitamin D <75 nmol/L [= 30 ng/mL] were substituted with 300000 IU cholecalciferol orally. 25OH-vitamin D, BSAP and crosslinks were controlled 3 month after supplementation. 25OH-vitamin D was corrected for circannual rythm $\left[y^{'}=y+17.875*\sin\;\left(2\frac{\pi }{365}*\mathrm{day}+2.06\right)\right]$[21], bone turnover markers were not corrected. According to the method published by Bolland who showed that the concentration of 25OH-vitamin D follows a sine curve, we corrected the values for: baseline + amplitude × sine (angular frequency × day of the year + phase shift). Angular frequency was 2 × π/365, and the phase shift is the translation from x axis. As the minimum/maximum concentrations were in february resp. august, phase shift was calculated accordingly. Amplitude was calculated from minimum and maximum monthly mean values (before supplementation).

No calcium supplementation or biphosphonate therapy was given. Paired students t-test was used to compare the two periods. Unpaired Students t test was used to compare 25OH-vitamin D levels between HIV and HCV/HBV patients.

Response to cholecalciferol was defined as ≥10% reduction in one or both of the two crosslinks. Calculation of factors that favour response was made by Chi-Square test for 2×2 tables. Correlations were made be linear regression with a significance level of 95%.

## Ethics approval

Treatment with cholecalciferol in patients with hypovitaminosis was considered as standard care, and decided by the attendant physician. So under Swiss regulations, this study did not need formal ethical approval.

## Results

### Differences between Hepatitis and HIV infected patients

**Figure 1:**
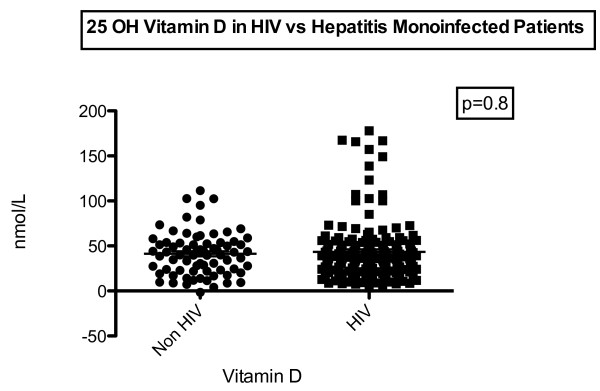
Differences between HIV and Hepatitis monoinfected patients.

25OH-vitamin D, BSAP and crosslinks were measured in 96 HIV infected patients. 25OH-vitamin D levels were compared with 70 HBV or HCV monoinfected patients. Baseline characters are shown in Tables 1 and 2. No difference in age between HIV and Hepatitis patients (42.5 ± 9.1 vs. 42.8 ± 11.2 years, p = 0.9) or gender was present (39/70 vs. 54/96 male patients, p = 0.9). 47/70 Hepatitis patients had an HCV infection. No significant difference in 25OH-vitamin D levels could be found between HIV and non HIV-patients (45.3 ± 29.7 vs. 48.8 ± 22.1 nmol/L, p = 0.4) (see Figure 1).

### Correlations between 25OH-vitamin D and bone turn over markers

No correlations between 25OH-vitamin D levels prior to substitution and bone turn over levels could be found. For BSAP, r value was -0.043 ± 0,045 p = 0.34. Also for bone resorption markers, no correlation was present with r value of 0.001 ± 0.12, p = 0.99 for PYR and -0.02 ± 0.03, p = 0.54 for DPD (See Additional file 1: Figure S1a).

### Substitution with cholecalciferol

**Figure 2:**
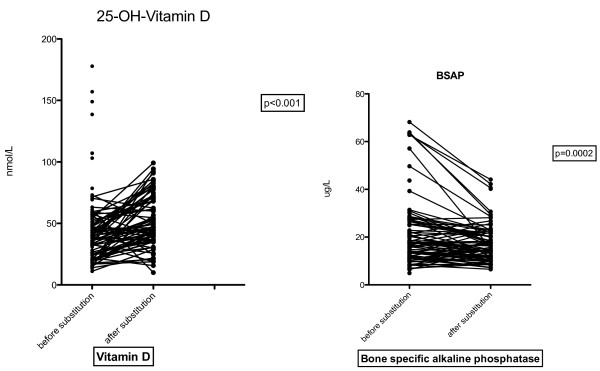
Vitamin D and BSAP before and after supplementation.

87/96 (90.6%) of the patients had a 25OH vitamin D level < 75 nmol/l, 10/96 (10.4%) were below 25 nmol/L. Four patients had an urgent indication for antiretroviral therapy and were not included for control after substitution, as ART can influence vitamin D as well as bone turn over markers. 6 patients did not provide urine samples 3 month after substitution. In 77 patients analysis of the effect of cholecalciferol substitution could be done. 3 month after substitution, corrected 25OH-vitamin D levels were significantly higher (42.7 ± 26.61 vs. 52.85 ± 21.8 nmol/L, p < 0.001). No patient had 25OH-vitamin D level in toxic range (>250 nmol/L) after substitution (See Figure 2).

### Bone turnover markers

Bone turnover markers fell significantly after substitutions. The bone formation marker BSAP fell from 21.31 ± 14.32 to 17.53 ± 8.17 μg/L (p < 0.001). Normal value for BSAP is < 14.3 μg/L pre- and 22.4 μg/L postmenopausal.

**Figure 3:**
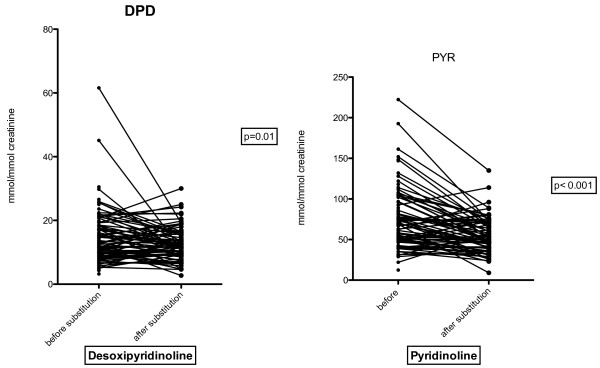
DPD and PYR before and after supplementation.

For bone resorption markers, PYR decreased from 74.57 ± 36.83 to 54.82 ± 21.43 nmol/mmol creatinine (p < 0.001) and DPD from 15.17 ± 8.34 to 12.61 ± 5.02 nmol/mmol creatinine (p = 0.01) (See Figures 2 and 3).

### Factors for response

We defined response to cholecalciferol as at least 10% reduction in either PYR or DPD. This is arbitrary but no data exist regarding response to cholecalciferol and bone turnover markers. 52/77 (67.5%)patients did respond. We then correlated response with gender, HCV-infection, antiretroviral treatment, TDF-, PI- and NNRTI use, age, african descent, BMI < 21, smoker and 25OH-vitamin D level < 30 nmol/L. Antiretroviral treatment was correlated with better response to cholecalciferol (OR 2.76, CI 1.58 – 4.53, p = 0.002), however the low number of patients without antiretroviral treatment limits the statistical value of this data. For other factors, no significance could be observed (see Table 3).

**Table 3:** Univariate analysis for response to vitamin D

	**Responder**	**Non responder**	**OR**	**CI**	**p-value**
**HCV**	25	8	1.59	0.78-3.23	0.18
**No HCV**	27	17			
**IVDU**	23	6	1.91	0.86-4.23	0.08
**No IVDU**	29	19			
**Treatment**	48	16	2.76	1.58-4.03	0.002
**No Treatment**	4	9			
**TDF**	36	13	1.61	0.85-3.04	3.04
**No TDF**	16	12			
NNRTI*	27	6	1.9	0.78-4.57	0.14
**PI**	19	10			
**Male**	28	14	0.76	0.4-1.46	0.65
**Female**	24	11			
**African descent**	12	8	1.34	0.68-2.61	0.4
**No african descent**	40	17			
**Age < 40**	18	10	0.85	0.44-1.64	0.21
**Age > 40**	34	15			
**BMI < 21**	7	2	1.52	0.43-5.4	0.48
**BMI > 21**	45	23			
**Smoker**	32	13	1.29	0.68-2.49	0.42
**Non smoker**	20	12			
**Vit D 30-75 nmol/L**	38	17	0.97	0.47-2.01	0.92
**Vit D < 30 nmol/L**	15	7			
**Total**	52	25			

*34/36 NNRTI patients received EFV.

## Discussion

Low bone mineral density (BMD) as well as insufficiency fractures are more common in HIV-patients compared to the general population [3,11,22]. Vitamin D deficiency is believed to be more frequent in HIV-patients [4,23]. We did not compare our patients with the general population, but with patients with a hepatitis B or C mono-infection and did not find a difference in vitamin D levels. Although it is known that Efavirenz can decrease vitamin D levels [8], life circumstances may be a more important factor for low vitamin D level than HIV-infection itself.

It is known that antiretroviral therapy increases bone turn over markers [9]. In accordance to Welz [8], we did not find a direct correlation between bone turnover markers and 25OH-vitamin D leves at baseline, but a significant decrease after supplementation. Results of cholecalciferol supplementation on bone turnover are conflicting. Contrary to our results, two recent publications showed an increase of BTM after high dose cholecalciferol supplementation [24,25], but these results were seen primarily in the first weeks after intervention, while at 16 weeks and one year, supplementation resulted in a decrease in bone turnover markers [26,27].We did not measure or supplement calcium intake. Different results on BTM could be due to different calcium uptake, as the positive effect of active 1, 25OH vitamin D results in an increased calcium uptake, while the direct effect of vitamin D on osteoclasts and osteoblasts will enhance bone resorption and decrease bone formation [28]. In HIV-negative patients, most studies have been done in high risk populations with combined calcium and cholecalciferol supplementation [29]. These studies show a benefit with a reduction in fracture rate of 20% in patients in whom a target value of > 50 nmol/L 25OH-vitamin D could be achieved [30]. It is suggested that 25OH-vitamin D levels should even be supplemented to >75 nmol/L [31]. Even if the results of studies regarding fractures rate in HIV-patients yielded conflicting results and the effect of cholecalciferol is uncertain [32-36], we actually suggest cholecalciferol supplementation. We observed a decrease in bone resorption as well as bone formation markers 3 month after supplementation. A correlation between bone formation and bone resorption markers has be observed in other studies too [9,37]. We hypothesize that vitamin D may limit bone resorption, leading to a decrease in compensatory bone formation.

We used a high dose of cholecalciferol as “pulse therapy”. This increase in 25OH vitamin D was significant but only about two third of that found in a recent study using 300000 IU intramuscularly [25]. This marked effect was also seen in another study from Iran [38] but our results were similar to a study from Australia [39]. High dose pulse therapy with cholecalciferol has previously been a regularly followed strategy but some authors advocate lower doses on daily base. Two rationales for daily or weekly cholecalciferol doses have been proposed. First, vitamin D has also an effect on muscle [40]. High levels of vitamin D could rapidly improve muscle function in frail patients in whom coordination is still reduced, leading to falls and fractures [41]. Secondly, vitamin D intoxication has been seen with pulse therapy [42]. Hypersensitivity to vitamin D has been correlated to mutations in CYP24A1 and these patients may have elevated vitamin D levels without supplementation [43]. As vitamin D had not been measured in most patients before supplementation, intoxication could be due to hypersensitivity rather than to high doses of cholecalciferol alone. As we only substituted patients with 25OH-vitamin D deficiency, we think it is safe to give 300000 IU orally every 6 months. No toxic effects of vitamin D were observed. Even if life expectancy in HIV patients has markedly improved, frailty is still not a issue in most HIV-infected patients [44]. We therefore believe that the effect on muscle function may not lead to more falls. However, the optimal strategy for cholecalciferol supplementation has to be defined. Even if we could observe an increase in 25OH-vitamin D levels, only about 20% of our patients did reach target value of 75 nmol/L. Similar results has been shown in HIV patients given pulse [45] or continues low dose (4000 IU daily) supplementation [46].

We defined a decrease of ≥ 10% of either one or both bone resorption markers as response to cholecalciferol. This is an arbitrary definition but no cut-off values have been described. Even if we could find a significant overall decline in bone turnover markers, only 52/77 patients had a decrease in bone turnover markers, while 25 had not. We could not find a correlation with traditional risk factors or specific antiretroviral drugs and the response to Vitamin D supplementation in the univariate analyisis. Due to the lack of significance in univariate analysis, it is in our view inappropriate to perform a multivariate analysis, as these results cannot be interpreted properly. The same problem applies to the treated vs. non treated HIV-patients. Given the low number of patients without ART, the statistical significance compared to treated patients should be interpreted with caution. In Non-HIV patients, the effect of cholecalciferol on bone turnover markers shows conflicting results. While some studies show no effect despite benefit on BMD [47], others did show an amelioration of bone turnover [48,49].

Our study has some limitations. It is a relatively small, open and not placebo controlled trial, and we did not measure calcium intake and sun exposure. Even if bone turnover markers are directly correlated with fracture rate independently of BMD [50], they do not substitute for the later. Thus far, we only showed a short term effect on bone metabolism, while ultimately reduced fracture rate is the primary endpoint of this medical intervention. Thus we propose larger, long term studies including DXA-scans and clinical events.

## Conclusion

In summary, we showed that supplementation with high dose pulse therapy with cholecalciferol in HIV-patients with low 25OH vitamin D level resulted in a decline of bone resorption as well as in bone formation markers. As it has been shown that bone resorption and formation are linked, we postulate a protective effect on bone structure by cholecalciferol substitution.

## Competing interests

The authors declare that they have no competing interests.

## Authors’ contributions

RJP was main investigator, contributed to the design of the study, data collection, data analysis and interpretation and writing; MR contributed to design of the study, the data interpretation and revision of the manuscript; JPR contributed to the design, to the performances of urine analyses and revision of the manuscript; MS and CF contributed to the design and revision of the manuscript. All authors read and approved the final manuscript.

## Pre-publication history

The pre-publication history for this paper can be accessed here:

http://www.biomedcentral.com/1471-2334/13/577/prepub

## Supplementary Material

Additional file 1: Figure S1a Correlation between Vitamin D and bone turnover markers. Click here for file
