# Supplementary material for: Per oral substitution with 300000 IU vitamin D (Cholecalciferol) reduces bone turnover markers in HIV-infected patients
Source: BMC Infect Dis. 2013 Dec 6;13:577. doi: 10.1186/1471-2334-13-577 (PMC4029316; doi:10.1186/1471-2334-13-577)
Supplement: Additional file 1: Figure S1a — Correlation between Vitamin D and bone turnover markers. [file 1471-2334-13-577-S1.doc]

Additional file 1: Figure S1a : Correlation between Vitamin D and bone turnover markers
